# High prevalence of *Strongyloides stercoralis* in school-aged children in a rural highland of north-western Ethiopia: the role of intensive diagnostic work-up

**DOI:** 10.1186/s13071-016-1912-8

**Published:** 2016-12-01

**Authors:** Aranzazu Amor, Esperanza Rodriguez, José M. Saugar, Ana Arroyo, Beatriz López-Quintana, Bayeh Abera, Mulat Yimer, Endalew Yizengaw, Derejew Zewdie, Zimman Ayehubizu, Tadesse Hailu, Wondemagegn Mulu, Adriana Echazú, Alejandro J. Krolewieki, Pilar Aparicio, Zaida Herrador, Melaku Anegagrie, Agustín Benito

**Affiliations:** 1 National Center of Tropical Medicine, Institute of Health Carlos III, Madrid, Spain; 2 Mundo Sano Foundation, Madrid, Spain; 3 Parasitology Service, National Centre for Microbiology, Institute of Health Carlos III, Madrid, Spain; 4 Service of Microbiology and Parasitology, Hospital La Paz-Carlos III, Madrid, Spain; 5 Department of Microbiology, Immunology and Parasitology, College of Medicine and Health Science, Bahir Dar University, Bahir Dar, Ethiopia; 6 Instituto de Investigaciones en Enfermedades Tropicales, Universidad Nacional de Salta, sede regional Orán, San Ramón de la Nueva Orán, Salta, Argentina; 7 Consejo Nacional de Investigaciones Científicas y Técnicas (CONICET), Buenos Aires, Argentina; 8 National School of Health, Institute of health Carlos III, Madrid, Spain

**Keywords:** Soil-transmitted helminths, *Strongyloides stercoralis*, Baermann technique, Molecular techniques, Neglected, Ethiopia

## Abstract

**Background:**

Soil-transmitted helminthiases (hookworms, *Ascaris lumbricoides* and *Trichuris trichiura*) are extremely prevalent in school-aged children living in poor sanitary conditions. Recent epidemiological data suggest that *Strongyloides stercoralis* is highly unreported. However, accurate data are essential for conducting interventions aimed at introducing control and elimination programmes.

**Methods:**

We conducted a cross-sectional survey of 396 randomly selected school-aged children in Amhara region in rural area in north-western Ethiopia, to assess the prevalence of *S. stercoralis* and other intestinal helminths. We examined stools using three techniques: conventional stool concentration; and two *S. stercoralis*-specific methods, i.e. the Baermann technique and polymerase chain reaction. The diagnostic accuracy of these three methods was then compared.

**Results:**

There was an overall prevalence of helminths of 77.5%, with distribution differing according to school setting. Soil-transmitted helminths were recorded in 69.2%. Prevalence of *S. stercoralis* and hookworm infection was 20.7 and 54.5%, respectively, and co-infection was detected in 16.3% of cases. *Schistosoma mansoni* had a prevalence of 15.7%. Prevalence of *S. stercoralis* was shown 3.5% by the conventional method, 12.1% by the Baermann method, and 13.4% by PCR, which thus proved to be the most sensitive.

**Conclusions:**

Our results suggest that *S. stercoralis* could be overlooked and neglected in Ethiopia, if studies of soil-transmitted helminths rely on conventional diagnostic techniques alone. A combination of molecular and stool microscopy techniques yields a significantly higher prevalence. In view of the fact that current control policies for triggering drug administration are based on parasite prevalence levels, a comprehensive diagnostic approach should instead be applied to ensure comprehensive control of helminth infections.

## Background

Soil-transmitted helminths (STH) affect more than 2 billion people world-wide [1] and rank among the most prevalent neglected tropical diseases (NTDs) in sub-Saharan Africa (SSA) [2]. Hookworm is widely distributed in both rural and urban areas. While *Ascaris lumbricoides* and *Trichuris trichiura* are irregularly distributed, they are mainly found in urban areas [3]. Significant efforts have been and are being made to control their impact in endemic countries, with the ultimate target being to eliminate morbidity by 2020 [4]. Preventive chemotherapy is the key strategy for achieving this goal [5]. The World Health Organization (WHO) recommends regular mass drug administration (MDA) among school-aged and preschool-aged children (SAC, pre-SAC) in areas where prevalence exceeds 20% [6]. In areas of co-endemicity, integrated MDA is the recommended approach, e.g. albendazole-praziquantel for controlling STH and schistosomiasis [4].

Strongyloidiasis, caused by *S. stercoralis*, is an NTD known to be endemic in tropical and subtropical areas worldwide, affecting up to 100 million people [7, 8]. However, data on its epidemiology suggest that is highly underreported [9, 10]. This fact is mainly linked to diagnostic challenges: traditional identification of eggs in stool has nil sensitivity for *S. stercoralis*, and a high number of infections thus go undetected [11, 12]. Its diagnosis relies on identification of larvae in stool, with the two most appropriate methods being the Baermann, Harada Mori and Koga agar plate culture techniques, even though their sensitivity is not optimal [13]. While highly sensitive molecular techniques have recently been developed [14, 15], there is still no gold standard. Accordingly, a combination of different techniques is the most useful approach for the detection of *S. stercoralis* larvae [7].

Ethiopia is the second largest country in SSA, with a population of almost 92 million people, 84% of which live in rural areas [16]. Preventable communicable diseases, e.g. pneumonia, diarrhoea, measles and rabies, are the country’s major health problems [17]. Data on the burden of NTDs are incomplete, yet onchocerciasis, trachoma, lymphatic filariasis, STH, schistosomiasis, leishmaniasis and podoconiosis are all prioritized for intervention [18]. Ethiopia has the third highest prevalence of hookworm, the second highest prevalence of *A. lumbricoides* and fourth highest prevalence of *T. trichiura* in SSA [19]. According to different studies, the prevalence of *S. stercoralis* in the country over the last 25 years has ranged from 0.6% to as a high as 17.4%, with the highest reported rate is related to HIV-infected patients and hospital-based studies [20, 21]. However, *S. stercoralis* is not included in control programs [18].

The objectives of this study were: (i) to assess the prevalence of *S. stercoralis* infection among SAC in a rural area of Ethiopia; and (ii) to explore the usefulness of a diagnostic approach based on the combination of two parasitological techniques (conventional stool concentration and the Baermann method) and a molecular technique.

## Methods

### Study area

The Amhara Region is a subtropical zone lying at a height of 1,900 m above sea level, in the north-western of Ethiopia. The region’s capital, Bahir Dar (population approximately 250,000), is centrally situated at the southern tip of Lake Tana. There is a central urban nucleus and a surrounding rural area, in a radius of about 30 km from the city centre [22]. There are three distinct seasons, i.e. a rainy season, from June to September; spring, from mid-October until the end of December; and a dry season, during the reminder of the year. In the rural area there are 16 primary schools, which are structured in eight grades, with pupil’s age ranging from 5 to 6 in grade one, to 13–14 years in grade eight.

### Study design and study population

**Fig. 1:**
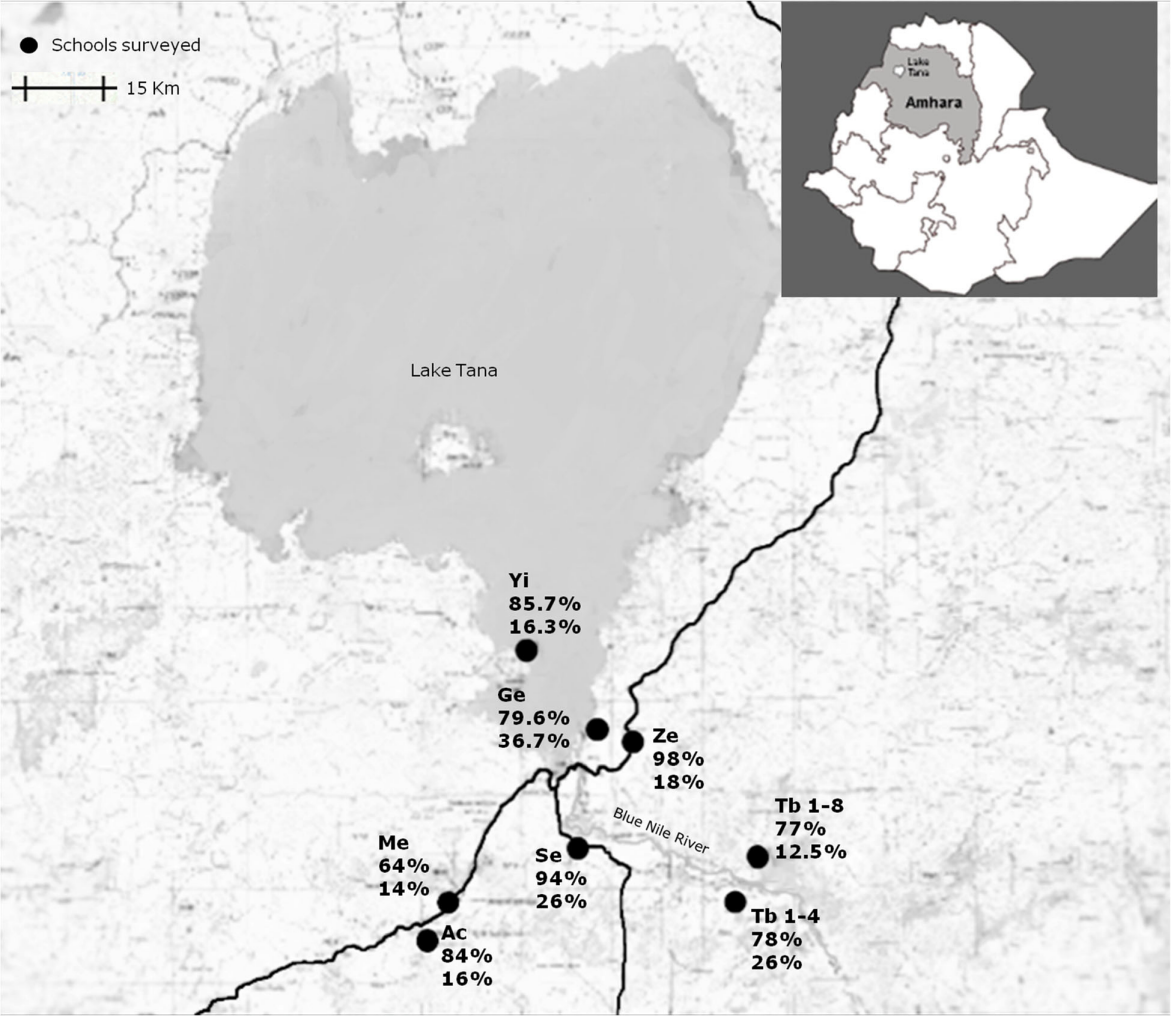
Location of the schools in the rural area of Bahir Dar. Overall prevalence of parasites (% at the *top*) and prevalence of *Strongyloides stercoralis* (% at the *bottom*) in each school. The map was created using Global Mapper v11.02. *Abbreviations*: Ac, Achadir; Me, Meshenti; Ge, Gedro; Ze, Zenzelema; Se, Sebatamit; Tb1-4, Tisabay 1-4; Tb 1-8, Tisabay 1-8; Yi, Yiganda)

We conducted a cross-sectional study targeting primary schools in the rural area of Bahir Dar from October to November 2013. Adequate sample size was computed by assuming a maximum prevalence of *S. stercoralis* of 5% [23], a marginal error of 2.5%, a 95% confidence interval, and a non-response rate of 10%. The list of schools was provided by the Amhara Regional Education Bureau. Sampling was carried out by random multistage stratified cluster strategy, with the primary sampling units being schools. To improve accuracy in the sample design, the schools were randomly selected with probability proportional to size. A total of eight schools were selected as follows: two in the south-west of the study area (Achadir and Meshenti); two in the north-east (Zenzelema and Gedro); three in the south-east, (Sebatamit, Tisabay 1–4 and Tisabay 1–8), and one in the north-west (Yiganda), on a small lakeside peninsula (Fig. 1). Secondly, we randomly selected children attending the first grades, i.e. one to four, in every school. The list of pupils was provided by the head teachers of the eight schools. Although a total of 46 pupils were selected from each school, 48 to 50 samples were collected in each case, yielding a final sample size of 396.

### Laboratory procedures

Stool samples were collected in a labelled container, without any preservation method. Samples were sent to the laboratory at room temperature, and processed within a maximum of four hours after collection.

### Parasitological examination

First, a formol ether concentration (FEC) of 0.5 g of stool was processed with the Bioparaprep MINI® system (Leti Diagnostics, Barcelona, Spain) according to the manufacturer’s instructions; the design of the system is based on a modification of Ritchie’s method, with filtering by the classical method been replaced by a filtration-concentration process [24]. Secondly, we applied the Baermann technique based on the active migration of larvae from fresh stool samples, i.e. when faeces are suspended in water, the larvae move into the water, sink to the bottom and can be collected for identification [25]. Samples were incubated for 18 h at 26 °C with activated charcoal before applying the Baermann technique. Every sample was checked three times by three different, experienced microscopists. Pathogenic helminth eggs/larvae and intestinal protozoa cysts were recorded. *Strongyloides stercoralis* larvae were identified by the buccal cavity and the genital organs in rhabditiform larvae, and the posterior tip in filariform larvae.

### DNA extraction

One aliquot of one g of stool sample was preserved in a container and stored at 4 °C for DNA extraction. A previous concentration of the sample with saline solution (0.9%) was prepared using a Bioparaprep MINI® device. DNA extraction, from 180 to 200 mg of the concentrated sample, was performed with a QIAamp® DNA stool mini kit (Qiagen, Hilden, Germany), following the manufacturer’s instructions. Previous procedures were performed in the School of Medicine laboratory at the University of Bahir Dar. For the PCR amplification, the DNA was sent to the department of Parasitology at the National Center of Microbiology (Institute of Health Carlos III, Madrid, Spain).

### DNA amplification

*Strongyloides stercoralis-*specific primers targeting the 18S ribosomal subunit, as described by Verweij et al. [26]: were used (Primer F: 5′-GAA TTC CAA GTA AAC GTA AGT CAT TAG C-3′; Primer R: 5′-TGC CTC TGG ATA TTG CTC AGT TC-3′). The amount of DNA was estimated with a nano-photometer, to ensure a concentration of five ng/μl in a final volume of 25 μl. A qualitative real-time polymerase chain reaction (RT-PCR) assay was performed, using a SybrGreen format (Invitrogen, San Diego CA, USA), as described by Saugar et al. [15]. Purified genomic DNA from *Strongyloides venezuelensis* L3 was used as positive control. The samples were assayed in duplicate. A third sample including 10 ng of *S. venezuelensis* DNA was also included as internal inhibition control. No template controls were included in each run. Amplification and detection were carried out using a Corbett Rotor-Gene™ 6000 RT-PCR cycler (Qiagen Corbett, Hilden, Germany) and data analysis in a Rotor Gene™ 6000 Series software version 1.7.

### Data and definition

We recorded participant’s gender, age and school grade. Infection by *S. stercoralis* was defined as positive when at least one of the three techniques was positive, i.e. presence of larvae in one/both parasitological test and/or positive PCR.

### Statistical analysis

We performed a descriptive analysis of parasitic infections using frequency tables; proportion and 95% confidence interval (CI) were used for the categorical variables while mean and standard deviation (SD) were used for the quantitative variables. Differences in parasite prevalence were assessed by the Chi-square test or Fisher’s exact test, and the association between infection and quantitative variables was analyzed by the *t*-test. The level of statistical significance was set at a value of *P* < 0.05. All statistical analyses were performed using the software package for statistical analysis, SPSS v.15.0.

## Results

The children had a mean age of 9.74 years (range 5–18 years; SD 1.91); 43% were female and 57% male. The gender and age distribution was similar across the total sample, at each school, and for the four school grades.

### Parasite prevalence

The prevalence of any intestinal pathogenic helminth or protozoan was 82.6% (95% CI: 78.9–86.3%), with the prevalence of helminths being higher than that of protozoans; at least one helminth was recorded in the 77.5% of the samples (95% CI: 73.2–80.4%) while at least one protozoan was recorded in 21.2% of samples (95% CI: 17.2–25.2%). Hookworm was the most frequent helminth, while the most frequent protozoan was *Giardia duodenalis*, recorded in 14.4% (95% CI: 11.1–17.9%), followed by *Entamoeba histolytica/dispar* detected in 9.1% (95% CI: 6.6–12.3%). Among positive samples, one parasite was observed in 55.4% (95% CI: 50–60.8%) and co-infection of two parasites in 31.5% of cases (95% CI: 26.5–36.5%). The location of the schools and the overall prevalence of parasites at each are summarized in Fig. 1. No association was found between age and infection.

### Helminths prevalence

Soil-transmitted helminths were recorded in 69.2% (95% CI: 64.5–73.5%). Hookworms were the most frequent, with a prevalence of 54.5% (95% CI: 49.6–59.4%). *Strongyloides stercoralis*, with a prevalence of 20.7% (95% CI: 17–25%), and *S. mansoni*, of 15.7% (95% CI: 12.1–19.3%), were the second and third most frequent, respectively. *Ascaris lumbricoides* and *Trichuris trichiura* were found in less than 10% of the children. Co-infection of hookworm and *S. stercoralis* was found in 16.3% (95% CI: 12.6–20.8%) and of hookworm and *S. mansoni* in 12.1% of the children (95% CI: 8.9–16.2%). Table 1 shows the prevalence of STH, *S. stercoralis* and *S. mansoni* in each area. Hookworms were the most prevalent in all the areas except in the north-west, were the prevalence of *A. lumbricoides* was the higher, being significantly different of the rest of the areas (*χ* ^2^ = 83.4, *df* = 1 *P* < 0.0001). *Hymenolepis* spp. were isolated in 5.8% (95% CI: 3.9–8.6%) of the students. *Diphyllobothrium latum* and *Trichostrongylus* spp. were detected one time each. No association was found between helminth infections and participant’s age.

**Table 1:** Overall prevalence of main intestinal parasites and prevalence in the four geographic areas

Overall (*n* = 396)														
Hookworm			*A. lumbricoides*			*T. trichiura*			*S. stercoralis*			*S. mansoni*		
Pos	%	95% CI	Pos	%	95% CI	Pos	%	95% CI	Pos	%	95% CI	Pos	%	95% CI
216	54.5	49.6–59.4	34	8.6	6.2–11.8	12	3.0	1.7–5.2	82	20.7	17.0–25.0	62	15.7	12.1–19.3
Geographic areas														
North-West (*n* = 49)				Pos	%	95% CI		North-East (*n* = 99)				Pos	%	95% CI
			Hookworm	17	35.0	21.4–48.0				Hookworm		71	71.7	62.2–79.7
			*A. lumbricoides*	21	43.0	29.0–56.7				*A. lumbricoides*		5	5.1	2.9–11.3
			*T. trichiura*	10	20.0	9.1–31.7				*T. trichiura*		0	–	–
			*S. stercoralis*	8	16.3	6.0–26.7				*S. stercoralis*		27	27.3	19.5–36.8
			*S. mansoni*	1	2.0	-1.9–6.0				*S. mansoni*		5	5.1	2.9–11.3
South-West (*n* = 100)				Pos	%	95% CI		South-East (*n* = 148)				Pos	%	95% CI
			Hookworm	49	49.0	39.4–58.7				Hookworm		79	53.4	45.4–61.2
			*A. lumbricoides*	4	4.0	1.6–9.8				*A. lumbricoides*		4	2.7	1.1–6.7
			*T. trichiura*	2	2.0	0.6–7.0				*T. trichiura*		0	–	–
			*S. stercoralis*	15	15.0	9.3–23.3				*S. stercoralis*		32	21.6	15.8–28.9
			*S. mansoni*	0	–	–				*S. mansoni*		56	37.8	30.4–45.9

### *Strongyloides stercoralis* prevalence

**Fig. 2:**
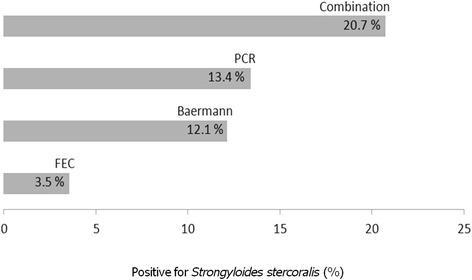
Prevalence (%) of *Strongyloides stercoralis* identified by each technique, and by the combination of the three techniques in the 396 samples

As a result of combining the three different techniques, 82 samples, 20.7% (95% CI: 17–25%), tested positive for *S. stercoralis.* Figure 1 shows the prevalence of *S. stercoralis* in each school. Infection varied across the four areas: *χ* ^2^ = 5.12, *df* = 1, *P* = 0.03 (Table 1). Not all stools shown to be *S. stercoralis-*positive by parasitological techniques were confirmed by PCR: 26.8% of the Baermann-positive and/or FEC-positive samples were confirmed by PCR (22/82), i.e. no *S. stercoralis* DNA was detected in 35.36% of positive samples (29/82). In contrast, 37.8% (31/82) were diagnosed by PCR alone. Figure 2 shows the prevalence of *S. stercoralis* in the 396 samples, as determined by individual techniques and by the combination of all three. PCR displayed higher sensitivity than the Baermann technique, *χ* ^2^ = 41.9, *df* = 1, *P* < 0.0001.

## Discussion

To our knowledge, 20.7% is the highest *S. stercoralis* prevalence figure reported in Ethiopia to date. The strength of our study lies in its use of a combination of parasitological and molecular techniques: 35% of the samples (29/82) were identified by parasitological techniques alone, and 37.8% (31/82) were identified by PCR. The final number of positive samples (82/396) relied on a combination of all three techniques. This approach, based on a combination of microscopic and molecular methods, had not been previously applied in the country. Among SAC, the highest previously reported prevalence was 3.4% in a study based on egg-detection techniques [23]. Moreover, our study area had been described as having a low prevalence of *S. stercoralis*, in that the most recent survey conducted in the Amhara Region reported a figure of 0.2% (five cases in 2,338 stool specimens) among children aged 2–15 years. What is more, FEC was the sole diagnostic technique used [27].

Integrated control programmes, e.g. for schistosomiasis and STH, have been launched in the country [28]. Our results indicate the need to include *S. stercoralis*. The prevalence of other STH could decline, while MDA with albendazole/mebendazole is ongoing but is unlikely to have an impact on *S. stercoralis.* Ivermectin is the treatment of choice [29, 30]. In countries where this has been introduced for control/elimination of onchocerciasis and lymphatic filariasis, prevalence of *S. stercoralis* has declined over time [31, 32].

The prevalence of hookworm in our sample is much higher than the prevalence reported by a local Amhara-based survey involving 2,338 children (9.7%) [27] and the estimated national prevalence of 16% [19]. This is probably linked to the poor sanitary conditions in the study area [33]. Such conditions make for the co-existence of and co-infection by both helminth species, since *S. stercoralis* has a similar transmission pattern [34].

The high prevalence of *A. lumbricoides* and *T. trichiura* on the peninsula in the north-west is noteworthy. The burden posed by *A. lumbricoides* and *T. trichiura* in Ethiopia is reportedly higher than that of hookworm, with the national average being estimated at 37% for *A. lumbricoides* and 30% for *T. trichiura* [19]. Low variation in temperature and environmental conditions in an isolated area such as the peninsula has been previously linked to these helminths infections [35].

The present study showed that *S. mansoni* and hookworm co-infections are common in SSA [36]; in our sample, *S. mansoni* displayed a clear pattern along the course of the river. Previous studies report a high transmission rate in the Lake Tana area at the end of the rainy season, exactly when our study was carried out. The low prevalence at the school on the peninsula has been accounted for by the displacement of the snail population - and the associated schistosome infections - towards the river in the rainy season, following the water flow [37].

The study has a number of limitations: (i) Using a single faecal sample may underestimate the prevalence of parasitic infections and fails to detect larvae in up to 70% [13]; analyses of samples over consecutive days would help to narrow this gap; (ii) *S. stercoralis* multiplies within the human host, establishing long-lasting infections [38]. Prevalence rates are often lower in children than in adults [39], and it can therefore be assumed that prevalence in the area could be much higher; (iii) PCR was the most sensitive technique in our analysis, while the amount of stool for the DNA extraction was one g; for the Baermann technique we used a minimum of 30 g. Intermittent elimination of larvae in stools, a low parasitic load, as well as other factors that can make DNA extraction heterogeneous must thus be explored. (iv) Applying PCR on the larvae isolate collected by the Baermann’s method would increase the PCR detection rate; also would confirm the detected larvae as of *S. stercoralis*, as well as the co-infection with hookworm; however, this was not applied in our study; (v) The higher prevalence in the eastern areas could be related with the proximity to the water, since the larvae thrive in warm wet soil. Even so, more local/behavioural factors should be analyzed.

## Conclusions

Our findings highlight the need for more studies to focus on the desirability of using a combination of methods for diagnosis of *S. stercoralis.* The information on the prevalence of infection rates suggests that *S. stercoralis* is highly underreported in SSA, owing to the fact that many studies often use low-sensitivity diagnostic methods, e.g. in our study, only 3.5% of the infections were diagnosed by FEC, whereas a figure of 20.7% was reached when techniques focusing on larvae detection were used in combination. National health policies in endemic countries must be supported by accurate ascertainment of STH epidemiology, including *S. stercoralis* and its related morbidity. Furthermore, our study data also support the possibility of routinely using a combination of albendazole and ivermectin during preventive chemotherapy campaigns targeting STH.

## Abbreviations

CI: Confidence interval
CONICET: Consejo Nacional de Investigaciones Científicas y Técnicas
DNA: Deoxyribonucleic acid
FEC: Formol ether concentration
MDA: Mass drug administration
NTDs: Neglected tropical diseases
pre-SAC: Pre-school-aged children
RICET: Red de Investigación Cooperativa de Enfermedades Tropicales
RT-PCR: Real-time polymerase chain reaction
SAC: School-aged children
SD: Standard deviation
SPSS: Software package for statistical analysis
SSA: Sub-Saharan Africa
STH: Soil-transmitted helminths
WHO: World Health Organization

## References

[1] Bethony J, Brooker S, Albonico M, Geiger SM, Loukas A, Diemert D, Hotez PJ (2006). Soil-transmitted helminth infections: ascariasis, trichuriasis, and hookworm. Lancet.

[2] Hotez PJ, Kamath A (2009). Neglected tropical diseases in sub-Saharan Africa: Review of their prevalence, distribution, and disease burden. PLoS Negl Trop Dis.

[3] Brooker S, Clements AC, Bundy DA (2006). Global epidemiology, ecology and control of soil-transmitted helminth infections. Adv Parasitol.

[4] WHO (2015). Investing to overcome the global impact of neglected tropical diseases: third WHO report on neglected tropical diseases.

[5] WHO (2012). Accelerating work to overcome the global impact of neglected tropical diseases-A roadmap for implementation.

[6] WHO (2012). Eliminating soil-transmitted helminthiases as a public health problem in children progress report 2001–2010 and strategic plan 2011–2020.

[7] Krolewiecki AJ, Lammie P, Jacobson J, Gabrielli AF, Levecke B, Socias E (2013). A public health response against *Strongyloides stercoralis*: time to look at soil-transmitted helminthiasis in full. PLoS Negl Trop Dis.

[8] Hotez PJ, Alvarado M, Basáñez MG, Bolliger I, Bourne R, Boussinesq M (2014). The global burden of disease study 2010: interpretation and implications for the neglected tropical diseases. PLoS Negl Trop Dis.

[9] Schär F, Giardina F, Khieu V, Muth S, Vounatsou P, Marti H, Odermatt P (2015). Occurrence of and risk factors for *Strongyloides stercoralis* infection in South-East Asia. Acta Trop.

[10] Bisoffi Z, Buonfrate D, Montresor A, Requena-Méndez A, Muñoz J, Krolewiecki AJ (2013). *Strongyloides stercoralis*: a plea for action. PLoS Negl Trop Dis.

[11] Montes M, Sawhney C, Barros N (2010). *Strongyloides stercoralis*: there but not seen. Curr Opin Infect Dis.

[12] Hotez PJ, Herricks JR (2015). Helminth elimination in the pursuit of sustainable development goals: a “worm index” for human development. PLoS Negl Trop Dis.

[13] Siddiqui AA, Berk SL (2001). Diagnosis of *Strongyloides stercoralis* infection. Clin Infect Dis.

[14] Becker SL, Piraisoody N, Kramme S, Marti H, Silué KD, Panning M (2015). Real-time PCR for detection of *Strongyloides stercoralis* in human stool samples from Côte d’Ivoire: diagnostic accuracy, inter-laboratory comparison and patterns of hookworm co-infection. Acta Trop.

[15] Saugar JM, Merino FJ, Martín-Rabadán P, Fernández-Soto P, Ortega S, Gárate T, Rodriguez E (2015). Application of real-time PCR for the detection of *Strongyloides* spp. in clinical samples in a reference center in Spain. Acta Trop.

[16] WHO African Region│Ethiopia. [http://www.who.int/countries/eth/en/]. Accessed 8 Sept 2016.

[17] Federal Ministry of Health. [http://www.moh.gov.et/home?p_p_auth]. Accessed 8 Sept 2016.

[18] Second edition of national neglected tropical diseases master plan 2015/16-2019/20. Addis Ababa: Federal Ministry of Health Ethiopia; 2016. https://www.google.es/search?q=Second+edition+of+national+neglected+tropical+diseases+master+plan+2015%2F16-2019%2F20.+Addis+Ababa.

[19] Deribe K, Meribo K, Gebre T, Hailu A, Ali A, Aseffa A, Davey G (2012). The burden of neglected tropical diseases in Ethiopia, and opportunities for integrated control and elimination. Parasit Vectors.

[20] Tadesse A, Kassu A (2005). Intestinal parasite isolates in AIDS patients with chronic diarrhea in Gondar Teaching Hospital, North-West Ethiopia. Ethiop Med J.

[21] Assefa T, Woldemichael T, Seyoum T (1991). Evaluation of the modified Baermann’s method in the laboratory diagnosis of *Strongyloides stercoralis*. Ethiop Med J.

[22] Bahir Dar City Profile. In: Tana high-level forum on security in Africa; 2015. [http://tanaforum.org/y-file-store/other_documents/bahir_dar_city_profile.pdf]. Accessed 8 Sept 2016.

[23] Abera B, Alem G, Yimer M, Herrador Z (2013). Epidemiology of soil-transmitted helminths, *Schistosoma mansoni*, and haematocrit values among schoolchildren in Ethiopia. J Infect Dev Ctries.

[24] Ritchie L (1948). An ether sedimentation technique for routine stool examination. Bulletin of the United States Army medical department.

[25] García LS, Bruckner DA (2001). Diagnostic medical parasitology.

[26] Verweij JJ, Canales M, Polman K, Ziem J, Brienen EA, Polderman AM, van Lieshout L (2009). Molecular diagnosis of *Strongyloides stercoralis* in faecal samples using real-time PCR. Trans R Soc Trop Med Hyg.

[27] King JD, Endeshaw T, Escher E, Alemtaye G, Melaku S, Gelaye W (2013). Intestinal parasite prevalence in an area of Ethiopia after implementing the SAFE strategy, enhanced outreach services, and health extension program. PLoS Negl Trop Dis.

[28] New NTD data to inform large-scale deworming in Ethiopia. [http://www3.imperial.ac.uk/newsandeventspggrp/imperialcollege/centres/sci/newssummary/news_15-7-2014-15-38-22]. Accessed 8 Sept 2016.

[29] Buonfrate D, Gobbi F (2016). Treatment of chronic *Strongyloides stercoralis* infection: moderate-to-low evidence shows that ivermectin is more effective and tolerable than albendazole and thiabendazole respectively. Evid Based Med.

[30] WHO (2015). Assessing the epidemiology of soil-transmitted helminths during a transmission assessment survey in the global programme for the elimination of lymphatic filariasis.

[31] Anselmi M, Buonfrate D, Espinoza AG, Prandi R, Marquez M, Gobbo M (2015). Mass administration of ivermectin for the elimination of onchocerciasis significantly reduced and maintained low the prevalence of *Strongyloides stercoralis* in Esmeraldas, Ecuador. PLoS Neggl Trop Dis.

[32] The Carter Center. Lymphatic filariasis elimination program. [http://www.cartercenter.org/countries/ethiopia-lymphatic-filariasis.html]. Accessed 8 Sept 2016.

[33] Tilahun SA, Collick AS, Ayele M. Water supply and sanitation in Amhara Region. Learning and communication Research Report, Bahir Dar, Ethiopia. 2012. [http://soilandwater.bee.cornell.edu/Research/international/docs/wateraid-finalreport_April10.pdf]. Accessed 8 Sept 2016.

[34] Echazú A, Bonanno D, Juarez M, Cajal SP, Heredia V, Caropresi S (2015). Effect of poor access to water and sanitation as risk factors for soil-transmitted helminth infection: selectiveness by the infective route. PLoS Negl Trop Dis.

[35] Karagiannis-Voules DA, Biedermann P, Ekpo UF, Garba A, Langer E, Mathieu E (2015). Spatial and temporal distribution of soil-transmitted helminth infection in sub-Saharan Africa: a systematic review and geostatistical meta-analysis. Lancet Infect Dis.

[36] Raso G, Vounatsou P, Singer BH, N’Goran EK, Tanner M, Utzinger J (2006). An integrated approach for risk profiling and spatial prediction of *Schistosoma mansoni*-hookworm coinfection. Proc Natl Acad Sci U S A.

[37] Erko B, Tedla S, Petros B (1991). Transmission of intestinal schistosomiasis in Bahir Dar, Northwest Ethiopia. Ethiop Med J.

[38] Puthiyakunnon S, Boddu S, Li Y, Zhou X, Wang C, Li J, Chen X (2014). Strongyloidiasis - an insight into its global prevalence and management. PLoS Negl Trop Dis.

[39] Schär F, Trostdorf U, Giardina F, Khieu V, Muth S, Marti H (2013). *Strongyloides stercoralis*: global distribution and risk factors. PLoS Negl Trop Dis.

